# Evaluation of the anti-inflammatory and antioxidant potential of the stem bark extract and some constituents of *Aidia genipiflora* (DC.) dandy (rubiaceae)

**DOI:** 10.1016/j.heliyon.2022.e10082

**Published:** 2022-08-13

**Authors:** Daniel Anokwah, Evelyn Asante Kwatia, Isaac Kingsley Amponsah, Yakubu Jibira, Benjamin Kingsley Harley, Elvis Ofori Ameyaw, Ernest Obese, Robert Peter Biney, Abraham Yeboah Mensah

**Affiliations:** a School of Pharmacy and Pharmaceutical Sciences, College of Health and Allied Sciences, University of Cape Coast, Cape Coast, Ghana; b Department of Pharmacognosy, Faculty of Pharmacy and Pharmaceutical Sciences, Kwame Nkrumah University of Science and Technology, Kumasi, Ghana; c Department of Pharmacology and Toxicology, School of Pharmacy and Pharmaceutical Sciences, University for Development Studies, Tamale, Ghana; d Department of Pharmacognosy, University of Health and Allied Sciences, Ho, Ghana

**Keywords:** *Aidia genipiflora*, Oleanonic acid, D-mannitol, Anti-inflammatory, Antioxidant

## Abstract

*Aidia genipiflora* (DC.) Dandy (Rubiaceae) is used to treat various microbial and inflammatory conditions by traditional healers in West African countries. However, there is no information on anti-inflammatory potential of *A. genipiflora*. This work therefore provides information on the anti-inflammatory and the antioxidant activities of the stem bark extracts and some bioactive constituents of *Aidia genipiflora.*

**Method:**

The anti-inflammatory activities of the extracts and compounds from *A. genipiflora* were investigated using the carrageenan-induced footpad oedema assay and the egg albumin denaturation assay. The antioxidant activities of the extract and compounds were investigated using the DPPH radical scavenging assay and the phosphomolybdenum total antioxidant capacity assay. The whole extract of *A. genipiflora* was also investigated for its acute oral toxicity using the fixed-dose procedure described by the Organization for Economic Cooperation Development guidelines.

**Result:**

The whole extract showed no acute toxicity effect and the LD_50_ was estimated to be greater than 3000 mg/kg body weight. The whole extract, methanol, and ethyl acetate fractions (30, 100, and 300 mg/kg) showed *in vivo* anti-inflammatory activity with respective percentage inhibition of oedema of 45.11 ± 3.41, 31.12 ± 3.42 and 29.28 ± 3.58 (p < 0.001) at the highest dose of 300 mg/kg. Diclofenac, used as a reference drug, gave a % inhibition of 48.94 ± 3.58. The compounds isolated from *A. genipiflora* demonstrated *in-vitro* anti-inflammatory activity at the IC_50_ range (16–96 μg/mL) compared to diclofenac (IC_50_ of 74.48 μg/mL). Oleanonic acid (AG1) and D-mannitol (AG4) further demonstrated *in vivo* anti-inflammatory activity (ED_50_ = 20.61 ± 1.29; 23.51 ± 1.26 mg/kg respectively) which was less potent compared to diclofenac (ED_50_ = 12.50 ± 1.41 mg/kg) in the carrageenan-induced oedema assay*.* The whole extract, pet. ether, ethyl acetate, and methanol fractions of *A. genipiflora* exhibited DPPH scavenging activities with respective IC_50_ of 222.2, 169.7, 121.5, and 40.7 μg/mL. The whole extract of *A. genipiflora* exhibited considerable total antioxidant capacity with respective values of 248.5 mg/g of ascorbic acid equivalent. All the compounds exhibited low DPPH scavenging activity with IC_50_ (64–86 μg/mL), compared to ascorbic acid (IC_50_ of 3.13 ± 1.20 μg/mL). These results highlight the anti-inflammatory and antioxidant activities of *Aidia genipiflora* stem bark extract and its constituents as evidence to support its traditional uses.

## 1 Introduction

*Aidia genipiflora* (DC.) Dandy, also referred as *Randia genipiflora* belongs to the family Rubiaceae. It is a hard-woody shrub or tree that is widely distributed in Sierra Leone, Ivory coast, Guinea – Bissau, Sudan, Cameroon and Ghana (Burkill, 1985), where it is commonly called ‘*otwensono*’ in Asante-Twi dialect in Ghana. The bark has been used for the management of dropsy, swelling, oedema and gout (stem, leaf) (Burkill, 1985). While there is evidence for the traditional use of the plant for inflammatory disorders (gout, oedema, swelling), no adequate scientific basis has been established.

Inflammation is an important defensive response of the body to noxious stimuli such as toxins, and pathogens. Though a protective mechanism, unregulated or exaggerated inflammation can induce or aggravate a number of diseases. It is documented that during inflammation, reactive oxygen species and free radicals are released which may trigger a series of chain reactions leading to prolonged healing (Khanna et al., 2014). The production of copious amounts of reactive oxygen species (ROS) during the inflammatory process has also been shown to underline the pathogenesis of many chronic disease conditions including rheumatoid arthritis, cancer, cardiovascular, and neurodegenerative diseases (Chen et al., 2018). Anti-inflammatory and antioxidant drugs are therefore pivotal in preventing and treating many human diseases. Unfortunately, the currently used anti-inflammatory drugs such as the Non-Steroidal Anti-inflammatory Drugs (NSAIDs) are associated with several adverse effects limiting their use (Bindu et al., 2020). As a result, the development of potent anti-inflammatory drugs with fewer side effects is required (Anokwah et al., 2016).

Therefore, plants or compounds with antioxidant activities could contribute to mitigating inflammatory disorder by mopping up reactive oxygen species and free radicals during inflammatory process. Preliminary data on the anti-inflammatory effects of the extracts were also been presented in our conference paper (Anokwah et al., 2019). The antimicrobial, anti-biofilm and efflux pump inhibition activities of the stem bark extracts and some constituents have been reported in our previous work (Anokwah et al., 2021). This study therefore seeks to provide additional information on possible anti-inflammatory and antioxidant effect of *A. genipiflora* and its bioactive constituents.

## 2 Materials and methods

### 2.1 Plant collection

The stem bark of *A. genipiflora* was collected from Kwahu Asakraka, a town in the Eastern Region of Ghana (06° 36.704′N/000° 42.659′W) in November, 2018. The plant material was authenticated by a botanist, Dr. George Henry Sam of the Department of Herbal Medicine, Kwame Nkrumah University of Science and Technology (KNUST), Kumasi, Ghana. A voucher specimen (KNUST/HM/2017/SB016) was kept at the herbarium of the faculty.

### 2.2 Drugs and chemicals

Dexamethasone (Pharm-Inter Brussels, Belgium), Diclofenac sodium (Troge Hamburg, Germany) and Carrageenan sodium (Sigma-Aldrich St. Louis, USA).

### 2.3 Preparation of extract and fractions

The whole extract (AG) and its pet. ether (AGPE), ethyl acetate (AGEt), and methanol (AGM) fractions were prepared as previously described by Anokwah et al. (2021). Three kilograms of the powdered stem bark was extracted by Soxhlet extraction with methanol/chloroform (4:1) for 6 h. The extract obtained was concentrated on a rotary evaporator under reduced pressure and temperature to obtain a brown solid extract with a yield of 5.1% w/w. About 100 g of the methanol/chloroform (4:1) whole extract was partitioned successively with petroleum ether (200 mL× 3), ethyl acetate (200 mL× 3), and methanol (200 mL× 3). The liquid extracts were concentrated on a rotavapor under reduced pressure and temperature and further dried in a hot air oven to afford solid extracts of pet ether (AGPE, 4.3 g), EtOAc (AGEt, 38.8 g), and MeOH (AGM, 53.2 g) fractions. The whole extract and fractions were kept in a desiccator until required for use.

### 2.4 Isolation and characterization of compounds

Chromatographic and spectroscopic techniques were used to obtain four isolates from the ethyl acetate fraction (AGEt) as previously described (Anokwah et al., 2021). About 29 mg of the ethyl acetate fraction was purified with open column chromatography with petroleum ether, ethyl acetate, and methanol by gradient elution. The eluates were monitored with thin layer chromatography and the resulting isolates were characterized by Nuclear Magnetic Resonance (NMR) and Mass Spectrometry (MS). The isolates were characterized as oleanonic acid (AG1), 4-hydroxy cinnamic acid docosyl ester (AG2), β-stigmasterol and β-sitosterol (AG3a:3b; 2:3) and D-mannitol (AG4).

### 2.5 Animals

Day-old chicks (*Gallus*; strain: Shaver 579) were procured from the Akate Farms (Kumasi, Ghana) whereas eight to twelve weeks old ICR mice were purchased from the Center for Scientific Research into Plant Medicine (mampong Akuapim, Ghana). The animals were kept in stainless steel cages of sizes; 34 cm × 57 cm x 40 cm in groups of 12 chicks per cage or 5 mice per cage. The chicks were fed with standard poultry feed (Chick Mash, GAFCO, Tema, Ghana) whereas the mice were fed with commercial rodent pellet (Agricare, Kumasi, Ghana). The feed and clean drinking water were provided *ad libitum*. The animal house temperature was kept at 28 ± 2 °C with a 12-hour light-dark cycle. This was ensured by using overhead incandescent illumination (Dickson et al., 2012). Day-to-day cage care was done with regular observation of the chicks/mice for weight and good health.

The procedures and techniques used for the *in vivo* experiment were as provided by the National Institute of Health guidelines for care and use of laboratory animals (Directive, 2010/63/EU; Animal Care and Use Committee, 1998) and was approved by the Committee in charge of animal studies (FPPS/PCOL/010/2018), Faculty of Pharmacy and Pharmaceutical Sciences, Kwame Nkrumah University of Science and Technology.

### 2.6 Acute oral toxicity assay

The whole extract of *A. genipiflora* was investigated for their acute oral toxicity using the fixed-dose procedure described by the Organization for Economic Cooperation Development (Organisation for Economic Co-operation and Development, 2008) guidelines. The dried extracts were reconstituted in 2% tragacanth saline solution (w/v). The extracts at fixed doses (100, 1000 and 3000 mg/kg) were administered orally to randomly chosen healthy groups of Institute of Cancer Research (ICR) mice (n = 5). The control group were given normal saline (0.9 %). The mice were denied food for 4 h before administration of the extracts and further denied food for 1 h after dosing to avoid drug-food interactions. The treated groups and control group of animals were monitored at 20 min intervals for the first 6 h and then intermittently for the first 24 h for signs and symptoms of overt toxicity. The animals were subsequently observed daily for 14 days.

### 2.7 Anti-inflammatory activity

#### 2.7.1 Carrageenan induced footpad oedema

The anti-inflammatory activities of the whole extract, fractions and isolated compounds from *A. genipiflora* were assessed using a curative protocol of the carrageenan-induced oedema in chick model (Roach and Sufka, 2003) with modifications (Anokwah et al., 2016). Inflammation was induced with carrageenan (100 μL of 2 % in saline, w/v) injected sub-plantar into the right footpads of the chicks after taking the baseline reading of the foot diameter for all the chicks. The whole extracts and fractions were reconstituted in 2% tragacanth saline solution (w/v) or 2% tween 80 saline solution (v/v) for compounds (AG1 and AG2) and given at different doses (30, 100, and 300 mg/kg *p. o*). Dexamethasone (1 mg/kg, *i. p*) and diclofenac (30 mg/kg, *i. p*) were used as positive controls. The vehicle for reconstitution (2% ^w^/_v_ tragacanth/tween 80 in 0.9% normal saline) was used as negative control and administered by mouth (p.o). The extracts and isolates were administered at 90 min by mouth (*p.o*) whereas the standard drugs were administered at 120 min by intraperitoneal injection (*i.p*) post carrageenan injection. The foot thickness was measured at intervals (0, 2, 3, 4, 5 and 6 h) using an electronic calliper (model: Z22855, Milomex Ltd., Bedfordshire, UK). Each test result was calculated as a mean of three repeated measurements.

#### 2.7.2 Inhibition of egg albumin denaturation assay

The *in vitro* anti-inflammatory activity of the compounds was investigated using the method described by Chandra et al. (2012) with modifications. The egg albumin was extracted from freshly laid hen eggs. Different concentrations of the isolated compounds and diclofenac) at concentrations (3.9–500 μg/mL) were prepared by serial dilution. The test tubes were filled with 5 mL of the reagent mixture which contained 0.2 mL of egg albumin, 2.8 mL of phosphate buffered saline (PBS, pH 6.4) and 2 mL of compound/standard drug reconstituted with 2% tween 80 in double-distilled water. Double-distilled water was used as the control (blank). The mixtures were mixed slowly by shaking the test tubes and incubated at 37 °C for 15 min and then kept in water bath at 70 °C for 5 min. The mixture was cooled and 200 μL pipetted into wells of 96-well plate and the absorbance was measured at 660 nm. The % inhibition of protein denaturation was estimated by considering denaturation in control as 100 % and calculated by using the formula:

% inhibition = 100 × (At/Ac - 1)

∗At = absorbance of test sample, Ac = absorbance of control sample.

### 2.8 Antioxidant activity

#### 2.8.1 2, 2-diphenyl-1-picrylhydrazyl (DPPH) free radical scavenging assay

The free radical scavenging activity of the extract/compounds were investigated using the DPPH scavenging assay was done in accordance with previously described protocol (Amponsah et al., 2013).

One millilitre each of the extracts reconstituted in methanol (at concentrations between 31.25 and 500 μg/mL) and compounds constituted in methanol (1.56–100 μg/mL) was mixed with 3 mL of DPPH (20 mg/L in methanol). The mixture was incubated in the dark at 25 °C for 30 min. The reaction mixture (200 μL) was pipetted into wells of 96-well plates and the absorbance was measured at 517 nm in a Cecil UV/VIS Spectrophotometer (Model: CE 7200, Milton, England). Methanol was used as the control (blank) and Ascorbic acid was used as the reference compound. The experiments were performed in triplicate and results presented as the mean ± SD of three values. The percentage free radical scavenging activity was calculated according to the following equation:

$$\text{\% ​DPPH ​scavenging ​activity ​}=\text{ ​}\frac{\text{Absorbance ​of ​sample}-\text{ ​Absorbance ​of ​control}}{Absorbance\;of\;control}\;\times 100$$

The IC_50_ for the extracts/compounds were calculated using GraphPad Prism for Windows version 6.0 (GraphPad Software, San Diego, CA, USA).

### 2.9 Total antioxidant capacity (TAC)

The total antioxidant capacity assay was done following previously described method (Prieto et al., 2013) with some modification. Different concentrations of the extracts (31.25–500 μg/mL) and the reference compound, ascorbic acid (3.125–100 μg/mL) were prepared using serial dilution approach. The phosphomolebdenum reagent was prepared with 0.6M H_2_SO_4_, 28mM Na_2_HPO_4_ and 4mM Ammonium molybdate. The extract (1 mL) and the phosphomolebdenum reagent (3 mL) were added and mixed in a test tube and incubated at 95 °C for 90 min. The reaction mixture was cooled to room temperature after the incubation period. The reaction mixture (200 μL) was pipetted into the wells of 96-well plate and the absorbance was measured at 695 nm. Methanol (1 mL) was used as the blank. The differential absorbances of ascorbic acid relative to the blank were used to plot a calibration curve of concentration against absorbance. All experiments were performed in triplicates and the TAC of the extract was expressed as Ascorbic acid equivalent (AAE) in mg/g of dried extract.

### 2.10 Statistical analysis

The statistical analysis was done using GraphPad Prism for Windows version 6.0 (GraphPad Software, San Diego, CA, USA) and P < 0.05 was considered as statistically significant. Oedema was quantified by measuring the differential foot thickness at each time point relative to time zero. The raw data for foot thickness were individually normalized as percentage change comparative to their corresponding values at time zero, and then averaged for the respective treatment groups. Treatment-time-course curves were drawn for the extracts/drugs and subjected to two-way (treatment × time) repeated measures analysis of variance with Bonferroni’s *post hoc t test*.

Total foot thickness for each treatment was calculated in arbitrary unit as the area under the curve (AUC). The percentage inhibition for each treatment was estimated using the equation;

$$\text{\% ​inhibition ​of ​oedema ​}=\text{ ​}\frac{AUC\;control\;–\;AUC\;treatment}{AUC\;control}\times 100$$

Differences in AUCs were analysed by ordinary one-way ANOVA; followed by Bonferroni’s *post hoc* test.

## 3 Results

### 3.1 Isolated compounds from *A. genipiflora*

**Figure 1:**
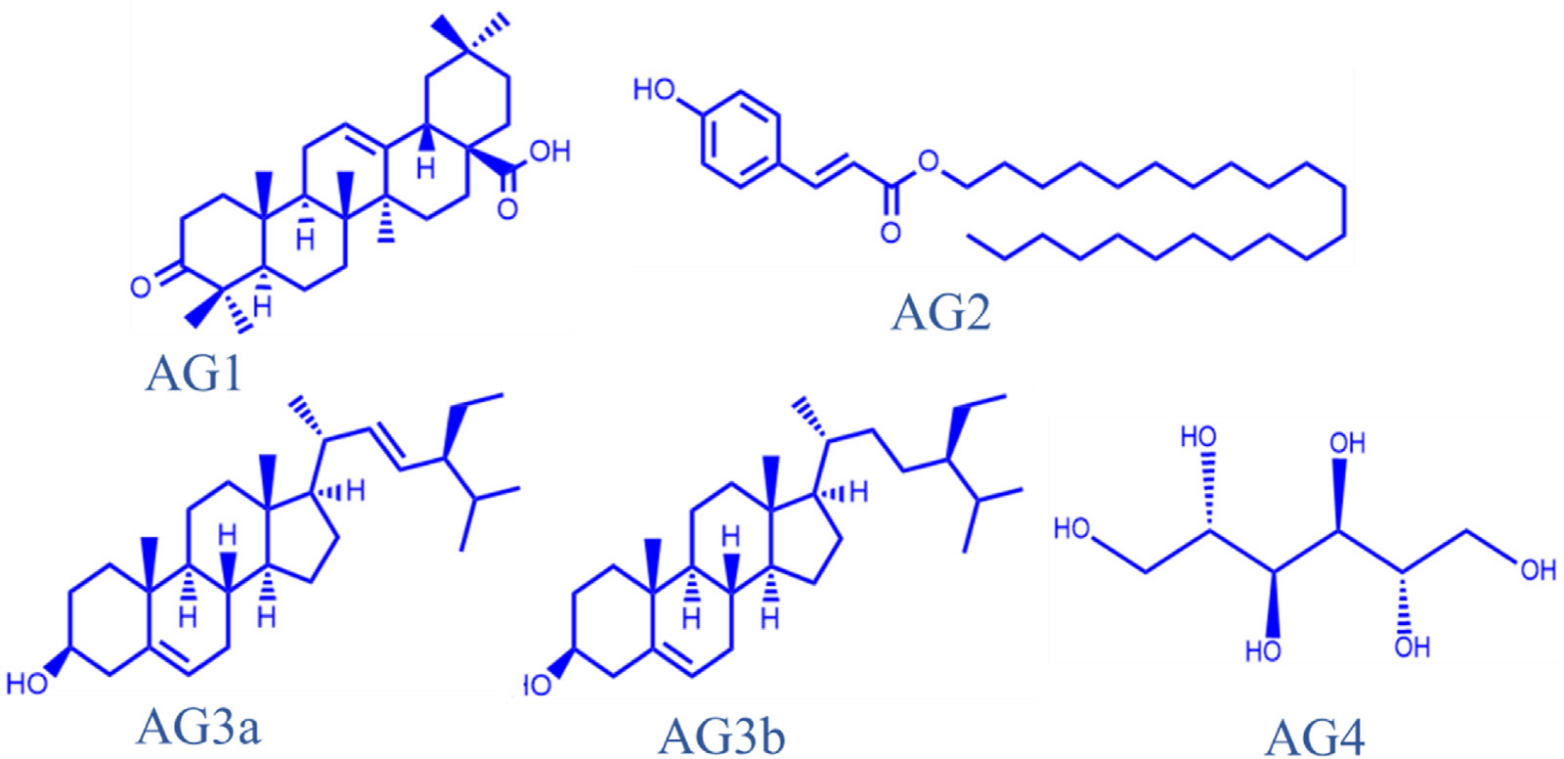
Isolated compounds from the stem bark of *Aidia genipiflora.*

Figure 1 shows the structures of oleanonic acid (**AG1**), 4-hydroxy cinnamic acid docosyl ester (**AG2**), β-stigmasterol and β-sitosterol (**AG3a/3b**; 2:1) and D-mannitol (**AG4**) which were isolated and characterized from the ethyl acetate fraction of *A. genipiflora* as reported in our previous work (Anokwah et al., 2021).

### 3.2 Acute oral toxicity effect

The whole extract of *Aidia genipiflora* was investigated for acute toxicity effect and the LD_50_ was estimated to be greater than 3000 mg/kg body weight. The extract showed no toxicity on the observed parameters (Table 1). All animals were healthy and active after 14 days following oral administration of the extract.

**Table 1:** Acute toxicity effect of *A. genipiflora.*

Observation	Control	100 mg/kg	1000 mg/kg	3000 mg/kg
Change in skin colour	Normal	Normal	Normal	Normal
Posture	Normal	Normal	Normal	Normal
Diarrhoea	Absent	Present	Present	Present
Seizures	Absent	Absent	Absent	Absent
Micturition	Normal	Normal	Normal	Normal
Drowsiness	Absent	Absent	Absent	Absent
Dyspnoea	Absent	Absent	Absent	Absent
Sedation	Absent	Absent	Absent	Absent
Fasciculation	Absent	Absent	Absent	Absent
Weight loss	No	No	No	No
Ability to feed	Normal	Normal	Normal	Normal
Death	No	No	No	No

### 3.3 Anti-inflammatory activity of the extract and fractions

**Figure 2:**
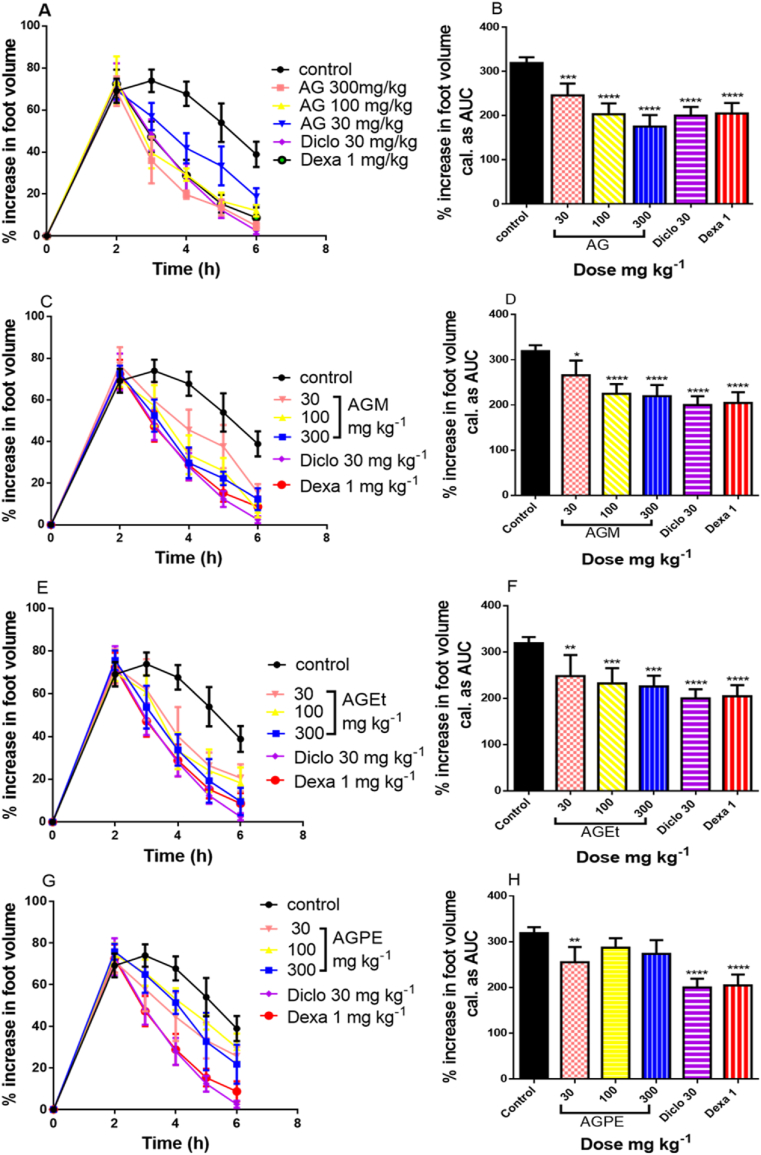
Effect of extracts (30–300 mg/kg *p. o.*), diclofenac (30 mg/kg *i. p.*) and dexamethasone (1 mg/kg *i. p*.) on the time course curves (A, C, E, G) and total oedema response (B, D, F, H) in carrageenan-induced foot oedema in chicks. Data is presented as mean ± S.E.M. (n = 5); ∗∗∗∗P < 0.0001; ∗∗∗P < 0.001; ∗∗P < 0.01, ∗P < 0.05 compared to vehicle-treated group (1-way ANOVA followed by Bonferroni’s post hoc test).

*A. genipiflora* whole extract and its fractions as well as the standard drugs (diclofenac and dexamethasone) showed significant inhibition (*p* < 0.05) effect on carrageenan-induced oedema in chicks (Figure 2). From the time-course curves (Figure 2 a, c, e, g), injection of 100 μL of 2 %^w^/v carrageenan sub-plantar resulted in inflammation which caused an increase in footpad volume peaking at about 2–3 h, after which a slow decrease in oedema was observed. The total oedema produced by each treatment was expressed in arbitrary units as the area under curve (AUC) of the time-course curves (Figure 2 b, d, f, h). The highest anti-inflammatory activity was exhibited by the whole extract (AG) which significantly (p < 0.001) reduced total oedema in a dose dependent manner with a percentage reduction of oedema of 45.11 ± 3.41%, 36.34 ± 3.83% and 22.79 ± 4.29% at 300, 100 and 30 mg/kg of the extract respectively. The MeOH (AGM) and EtOAc (AGEt) fractions also reduced total oedema in a dose dependent manner with a highest inhibition of 31.07 ± 3.49% and 29.28 ± 3.58% respectively at the highest dose of 300 mg/kg. The anti-inflammatory effect of the pet ether fraction (AGPE) was the lowest among the test samples and not dose-dependent. The positive controls diclofenac and dexamethasone demonstrated a slightly higher suppression of foot oedema than AGM, AGEt and AGPE fractions but had a similar effect as the whole extract. The percentage reduction of total foot oedema for the extract treated groups is presented on Table 2.

**Table 2:** Percentage inhibition of carrageenan-induced foot oedema.

Sample	Dose (mg/kg)			
	1	30	100	300
AG	-	22.79 ± 4.29∗∗∗	36.34 ± 3.83∗∗∗∗	45.11 ± 3.41∗∗∗∗
AGM	-	16.85 ± 3.61∗	29.33 ± 3.61∗∗∗∗	31.07 ± 3.49∗∗∗∗
AGEt	-	22.33 ± 5.98∗∗	27.20 ± 4.48∗∗∗	29.28 ± 3.58∗∗∗
AGPE	-	20.08 ± 3.71∗∗	9.70 ± 4.26	14.09 ± 4.73
Diclofenac	-	37.17 ± 3.32∗∗∗∗	-	-
Dexamethasone	35.70 ± 3.92∗∗∗∗	-	-	-

Key: Data is presented as mean ± S.E.M. (n = 5); ∗P < 0.05; ∗∗P < 0.01; ∗∗∗P < 0.001; ∗∗∗∗P < 0.0001; compared to vehicle-treated group (1-way ANOVA followed by Bonferroni’s post hoc test).

### 3.4 Anti-inflammatory activity of the isolated compounds

#### 3.4.1 *In vitro* anti-inflammatory effect of compounds

**Figure 3:**
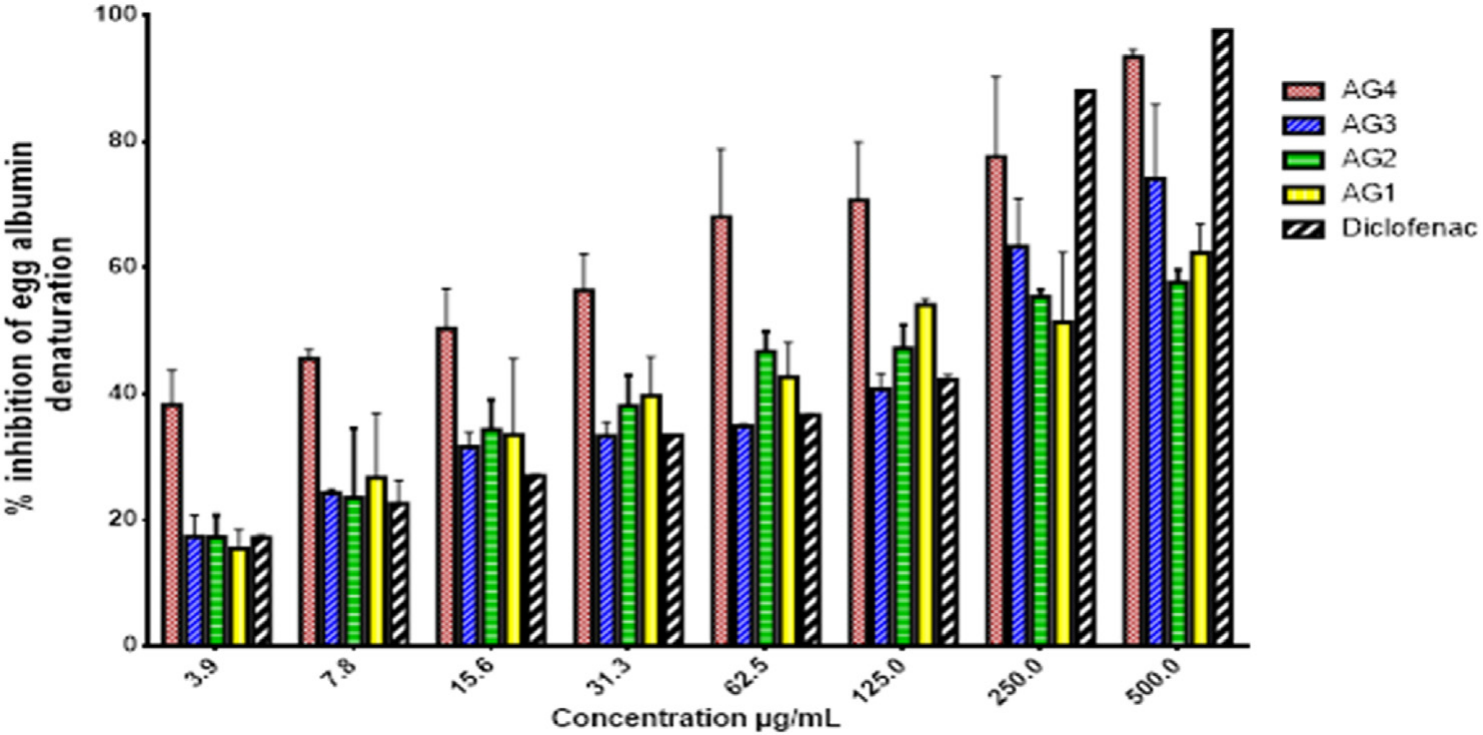
Effect of isolated compounds at concentration range (3–500 μg/mL) on protein denaturation expressed as percentage inhibition of egg albumin denaturation.

The compounds oleanonic acid (AG1), 4-hydroxy cinnamic acid docosyl ester (AG2), mixture of β-stigmasterol and β-sitosterol (AG3) and D-mannitol (AG4) were investigated for anti-inflammatory activity using egg albumin denaturation inhibition assay. All the compounds showed concentration dependent activity (Figure 3). The effects were expressed as IC_50_ (Table 3).

**Table 3:** Egg albumin denaturation inhibition assay.

Compound	AG1	AG2	AG3	AG4	Diclofenac
IC_50_ (μg/mL)	95.97 ± 1.43	102.36 ± 1.44	111.90 ± 1.32	16.88 ± 1.34	74.48 ± 1.31

#### 3.4.2 *In vivo* anti-inflammatory activity of compounds

**Figure 4:**
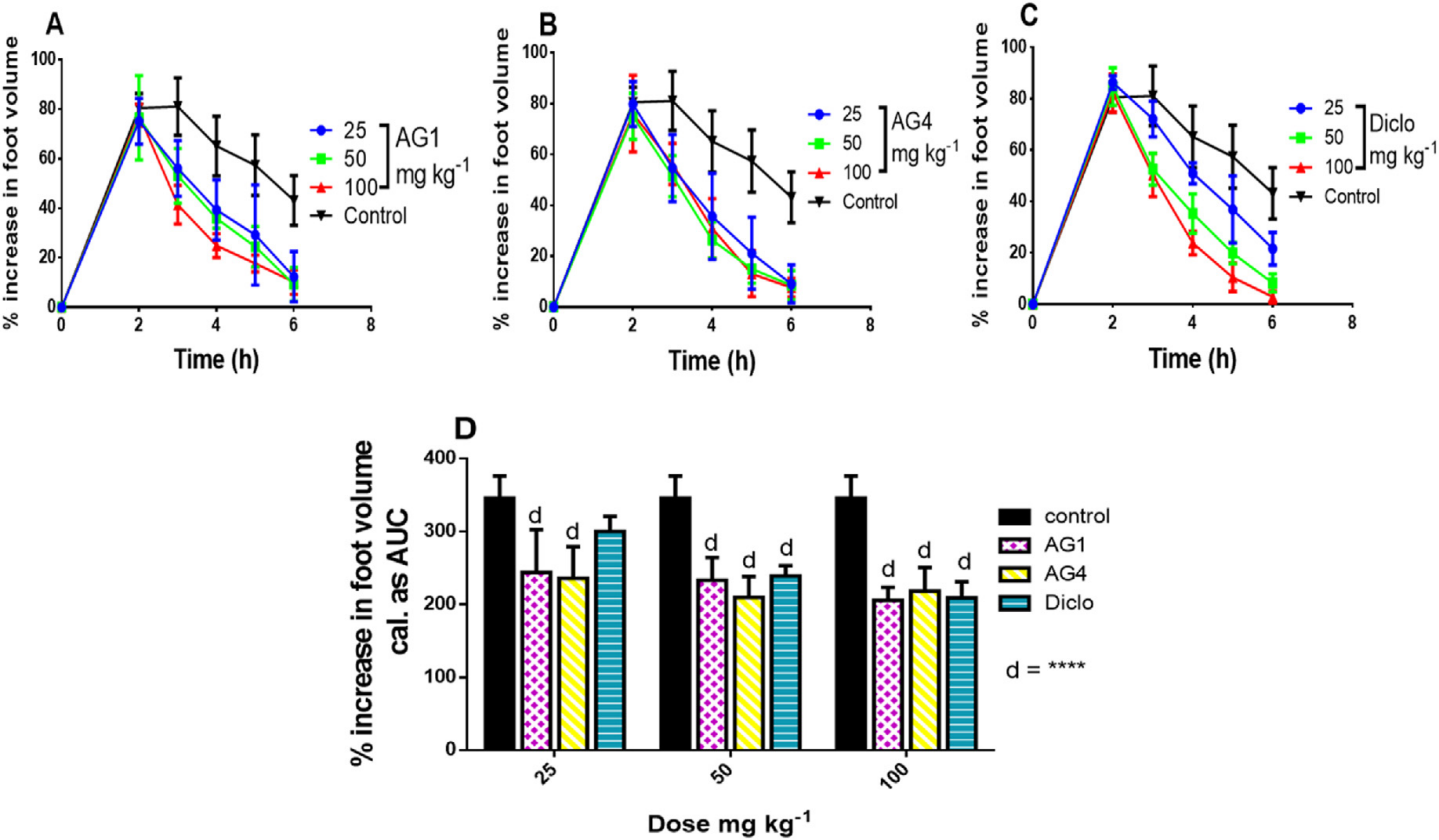
Effect of compoundsAG1 and AG4 (25–100 mg/kg p. o.) and diclofenac (25–100 mg/kg *i. p*.) on the time course curves (A, B and C) and total oedema response (D) in carrageenan-induced foot oedema in chicks. Data is presented as mean ± S.E.M. (n = 5); ^d^ P < 0.0001 compared to vehicle-treated group (1-way ANOVA followed by Bonferroni’s post hoc test).

The compounds AG1 and AG4 showed significant (p < 0.0001) effect on carrageenan induced oedema in chicks. From the time-course curves (Figure 4 A, B and C), injection of 100 μL of 2 %w/v carrageenan sub-plantar resulted in inflammation which caused an increase in footpad volume peaking at about 2–3 h, after which a gradual decrease in oedema was observed. The total oedema produced by each treatment was expressed in arbitrary units as the area under the curve (AUC) of the time-course curves (Figure 4 D). Compound (AG1 and AG4) demonstrated significant (p < 0.0001) reduction of total oedema at all doses (Table 4). The ED_50_ for AG1, AG4 and diclofenac were calculated as 20.61 ± 1.29 mg/kg, 23.51 ± 1.26 mg/kg and 12.50 ± 1.41 mg/kg respectively.

**Table 4:** Percentage inhibition of carrageenan-induced foot oedema.

Dose (mg/kg)	AG1	AG4	Diclofenac
25	29.66 ± 7.20∗∗∗∗	31.56 ± 6.15∗∗∗∗	13.15 ± 1.19
50	32.10 ± 5.14∗∗∗∗	39.17 ± 4.13∗∗∗∗	30.58 ± 3.13∗∗∗∗
100	40.00 ± 3.87∗∗∗∗	36.28 ± 5.58∗∗∗∗	39.37 ± 3.10∗∗∗∗

Key: Data is presented as mean ± S.E.M. (n = 5); ∗∗∗∗P < 0.0001; compared to vehicle-treated group (1-way ANOVA followed by Bonferroni’s post hoc test).

### 3.5 Antioxidant activity

#### 3.5.1 Antioxidant activity of the extracts and fractions

**Figure 5:**
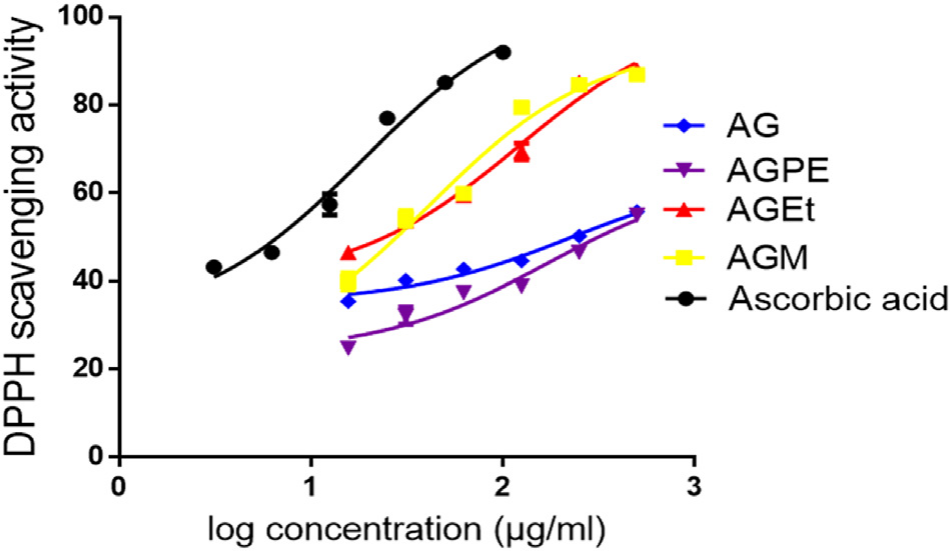
Percentage DPPH scavenging activity against log concentration of whole extract (AG), methanol (AGM), ethyl acetate (AGEt) and petroleum ether (AGPE) fractions of *A. genipiflora* extract and Ascorbic acid.

The extract and major fractions (Pet. Ether, EtOAc and MeOH) of *A. genipiflora* stem bark showed a concentration dependent radical scavenging effect (Figure 5). The fractions showed higher activities than the whole extract as indicated by the IC_50_ (Table 5).

**Table 5:** DPPH radical scavenging effect of *A. genipiflora* extracts expressed as IC_50_.

Crude extract or drug	IC_50_ (μg/mL)
AG	222.2 ± 1.3
AGPE	169.7 ± 1.4
AGEt	121.5 ± 1.2
AGM	40.7 ± 1.3
Ascorbic acid	18.6 ± 1.2

AG-whole extract; AGM- MeOH fraction; AGEt- EtOAc fraction; AGPE-pet-ether fraction.

**Figure 6:**
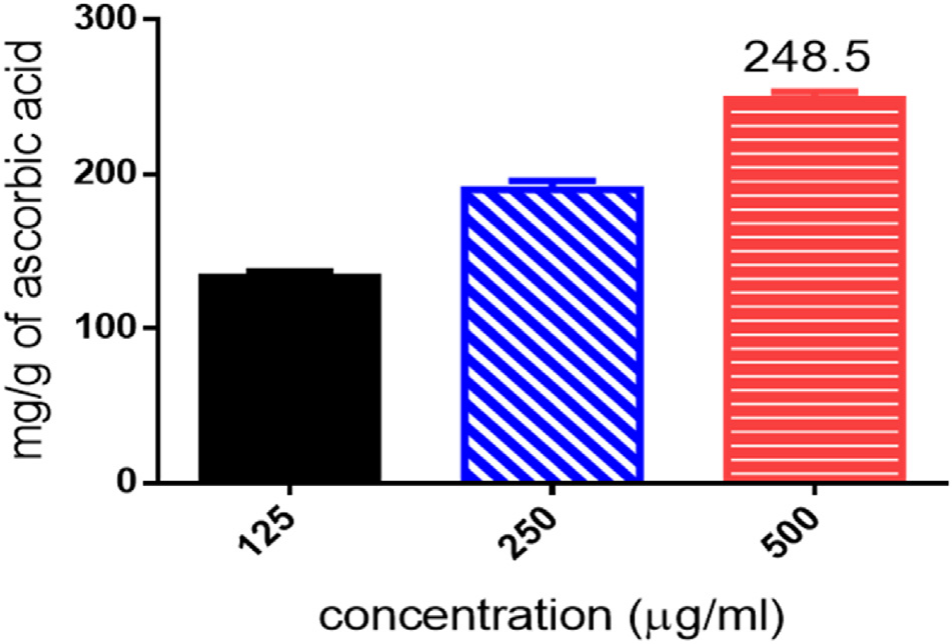
Standard calibration curve for ascorbic acid.

**Figure 7:**
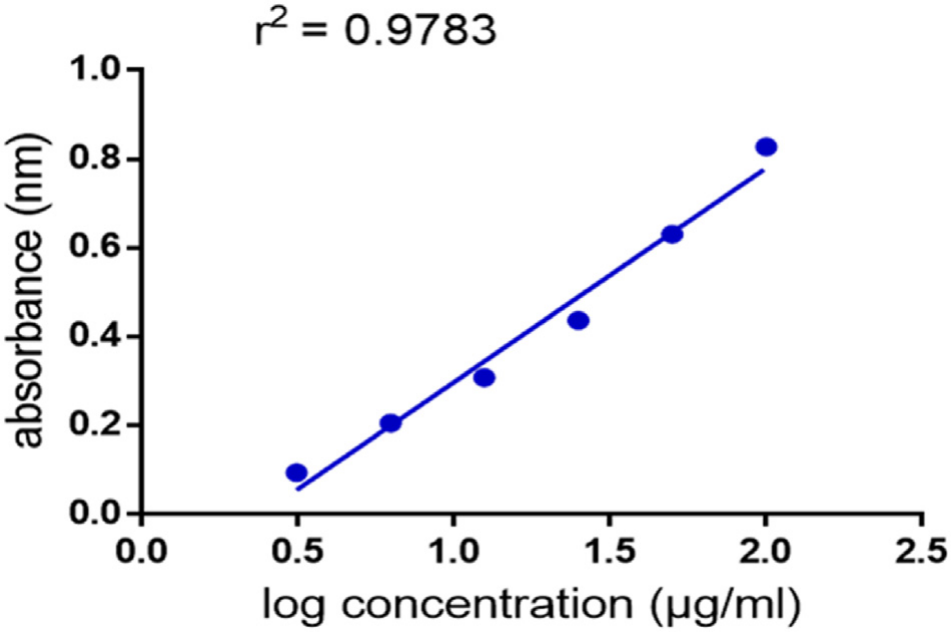
TAC of different concentrations of AG expressed as ascorbic acid equivalent.

The extract showed increasing total antioxidant capacity (TAC) with increasing concentration (Figure 6). The standard curve equation for ascorbic acid (Figure 7) was; y = 0.4813∗X – 0.1828, r^2^ = 0.9783. The total antioxidant capacity was determined to be 248.5 ± 2.64 mg g^−1^ dry weight of the extract (Ascorbic acid equivalent). This implies that a gram of the extract would have an antioxidant capacity equivalent to about 248 mg of Ascorbic acid.

#### 3.5.2 Antioxidant activity of the isolated compounds

**Figure 8:**
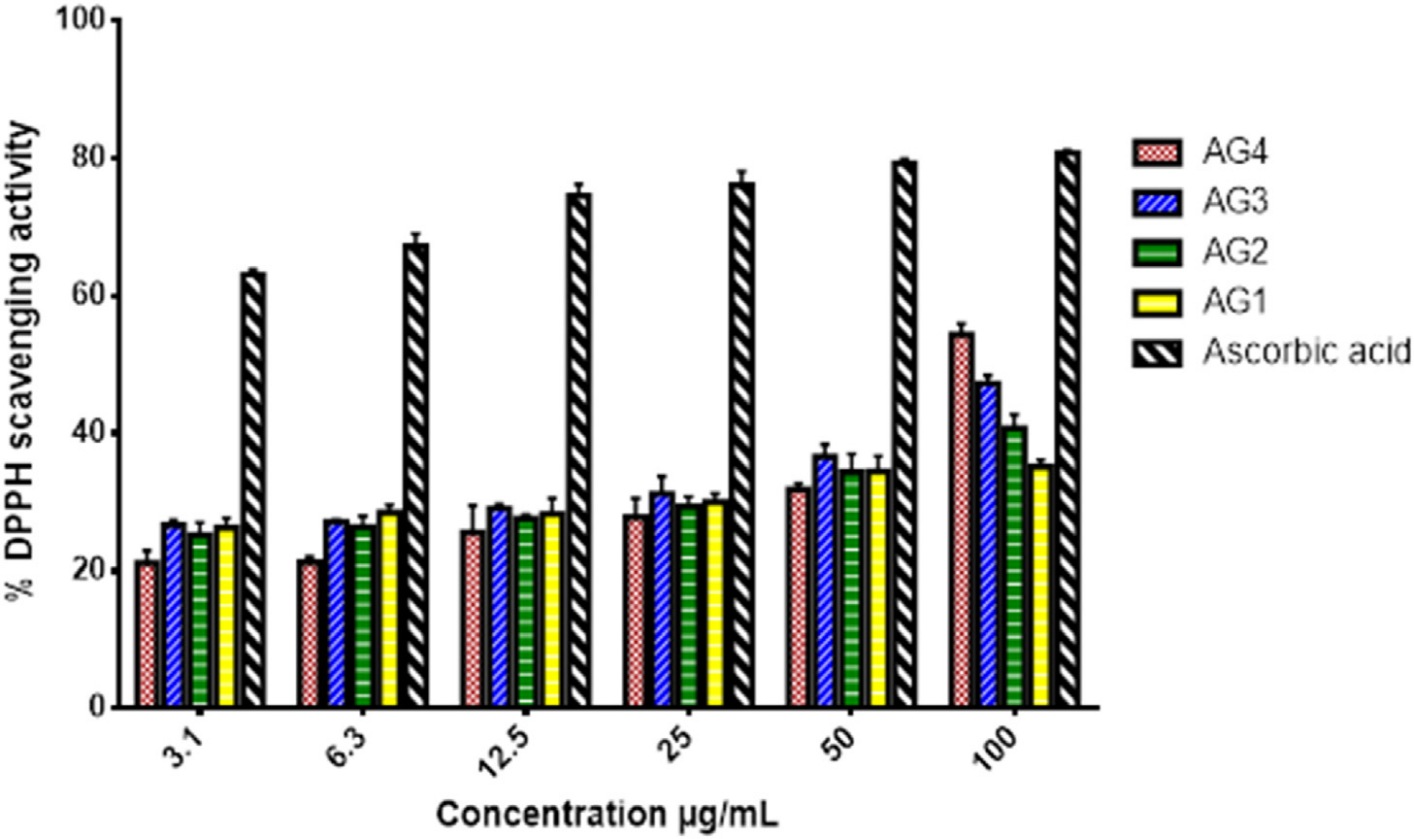
DPPH free radical scavenging effect of the isolated compounds.

The compounds isolated from the stem bark extract of *A. genipiflora* showed moderate DPPH radical scavenging activity (Figure 8) with IC_50_ range (60–80 μg/mL) compared to the activity of ascorbic acid (IC_50_ = 3.12 ± 1.20) (Table 6).

**Table 6:** DPPH radical scavenging effect of isolated compounds.

Compound	AG1	AG2	AG3	AG4	Ascorbic acid
IC_50_ (μg/mL)	85.67 ± 1.28	79.33 ± 1.24	64.84 ± 1.22	70.59 ± 1.16	3.13 ± 1.20

## 4 Discussion

The stem bark of *Aidia genipiflora* is used traditionally for the treatment of microbial infections and inflammatory conditions such as gout and oedema by traditional healers in some African countries. This study investigated the anti-inflammatory and antioxidant activity of *A. genipiflora* stem bark methanol-chloroform (4:1) whole extract, three solvent fractions (i.e., pet-ether, EtOAc and MeOH fractions) and some isolated constituents in animal and *in-vitro* models. The whole extract of the *A. genipiflora* stem bark had LD_50_ greater than 3000 mg/kg body weight which implies that the extracts had good safety profile for acute oral administration in accordance with the guideline 425 as suggested by the OECD guideline (Organisation for Economic Co-operation and Development, 2008). No mortalities were recorded after 14 days of monitoring and no decrease in the activeness or body weight of the animals was seen. There was diarrhoea observed in some of the animals given AG at all the doses (100, 1000, and 3000 mg/kg) administered within the initial 12 h after administration. D-mannitol has been reported to possess side effects of flatulence and diarrhoea (Ajaj et al., 2004), therefore suggesting that its presence in the extract could be the potential cause of the diarrhoea observed.

The anti-inflammatory activity of the extract and fractions of *A. genipiflora* were assessed using the carrageenan-induced footpad oedema in chick model. Oral administration of the extracts at doses 30, 100, and 300 mg/kg given within 90 min after inducing inflammation, resulted in significant anti-inflammatory activity of the whole extract, MeOH, EtOAc, and Pet. Ether fractions of both plants. The carrageenan-induced oedema has been described as a bi-phasic process involving an initial phase (1–3 h) characterized by the release of histamine, serotonin and bradykinin and a latter phase of swelling (3–6 h) characterized by the release of prostaglandins and cyclooxygenase (COX-2) (Necas and Bartosikova, 2013). Though the exact mechanism of anti-inflammatory action is unknown, the percentage inhibition of total oedema over the 6 h-period of observation suggests that the extracts may have inhibited the release or action of one or more of these inflammatory mediators. From the results, the whole extract of *A. genipiflora* (AG) had a higher anti-inflammatory effect than its MeOH (AGM), EtOAc (AGEt), and petroleum ether (AGPE) fractions implying a possible synergistic effect of constituents in the whole extract that were lost during fractionation (Ebelle-Etame et al., 2018). Among the fractions, AGEt and AGM showed similar anti-inflammatory activity better than AGPE.

Several compounds isolated from various classes of plant secondary metabolites including flavonoids, terpenoids, tannins, alkaloids, phytosterols and coumarins have demonstrated significant anti-inflammatory activities both *in vitro* and *in vivo* (Ghasemian et al., 2016)*.* The compounds isolated from the plant extract belong to some of these classes of secondary metabolite and could be responsible for the activity of the extracts. Therefore, compound AG1 (triterpenoid) and AG4 (hexitol) isolated in sufficient quantities from *A. genipiflora* were also investigated for anti-inflammatory activity using the carrageenan-induced oedema models. The compounds from *A. genipiflora*, AG1 and AG4, demonstrated anti-inflammatory activity with ED_50_ of 20.61 mg/kg and 23.51 mg/kg respectively while the standard drug, diclofenac had an ED_50_ of 12.50 mg/kg. The result implies that oleanonic acid and D-mannitol contribute to the anti-inflammatory activity of *A. genipiflora*. The result is in agreement with previous report by Giner-Larza et al. (2001) on the anti-inflammatory activity of oleanonic acid (AG1) which was demonstrated by the inhibition of bradykinin- and phospholipase A2-induced paw oedema, 12-O-tetradecanoyl-13-acetate (TPA)-induced dermatitis and 12-deoxyphorbol-13-phenylacetate (DPP)-induced ear oedema.

The *in vitro* anti-inflammatory activity of the isolated compounds was also investigated using egg albumin denaturation inhibition assay. Protein denaturation occurs when a variety of physical and chemical agents alter the electrostatic force, hydrophobic bonds, disulphide and hydrogen bonds in proteins, rendering them insoluble (Sangeetha and Vidhya, 2016). Denaturation of protein constituents within cells and intracellular substances are correlated with tissue injury, thus leading to inflammation (Osman et al., 2016). Therefore, the ability of a drug or compound to inhibit protein denaturation is apparently considered as potential for anti-inflammatory activity (Osman et al., 2016). Protein denaturation is mostly involved with chronic inflammation such as rheumatoid arthritis where denatured proteins act as auto-antigens leading to auto-immune disease (Karthik et al., 2013). NSAIDS such as diclofenac used in managing arthritic conditions are reported to inhibit protein denaturation (Karthik et al., 2013). Therefore, the protein denaturation inhibition activity of the compounds was investigated using diclofenac as the standard.

The result of the egg albumin denaturation inhibition assay revealed that all the compounds from *Aidia genipiflora* exhibited anti-inflammatory activity by significantly inhibiting protein denaturation compared to diclofenac. Among the isolated compounds from *A. genipiflora*, D-mannitol (AG4) showed the highest activity with an IC_50_ of 16.9 μg/mL which was about 4 times lower than an IC_50_ of 74.5 μg/mL for diclofenac (Table 3). Oleanonic acid (AG1), 4-hydroxy cinnamic acid docosyl ester (AG2) and the β-stigmasterol and β-sitosterol mixture (AG3a/b) demonstrated good activity with IC_50_s about 1.2–1.5 times higher than the IC_50_ for diclofenac. Previous reports show that β-stigmasterol and β-sitosterol have anti-inflammatory activity in acute anti-inflammatory rodent models (Paniagua-Pérez et al., 2017) and immuno-modulatory effect *in vivo* pig immune response model (Fraile et al., 2012).

The results of the anti-inflammatory assays indicated that AG1, AG2, AG3 and AG4 have potent anti-inflammatory activity. The result has provided scientific evidence to support the anti-inflammatory effect of *A. genipiflora* in managing inflammatory conditions by some traditional healers.

The antioxidant activities of the whole extract and fractions of *A. genipiflora* were assessed using the DPPH radical scavenging assay. From the results, the whole extract has low radical scavenging activity with an IC_50_ of 222.2 μg/mL which was about 12 times higher than the IC_50_ of ascorbic acid (IC_50_ of 18.6 μg/mL). Similarly, the ethyl acetate fraction and petroleum ether fractions showed low radical scavenging activities with respective IC_50_s about 7 times and 10 times higher than that of Ascorbic acid. The methanol fraction showed a potent radical scavenging activity with an IC_50_ of 40.7 μg/mL which was about two times higher than the IC_50_ of ascorbic acid (Table 4.9).

The whole extract, AG was also evaluated for its total antioxidant capacity using the phosphomolybdenum assay. The result of the total antioxidant capacity assay revealed that one gram of the whole extract will have similar antioxidant capacity as 248.5 mg of ascorbic acid, suggesting that the overall antioxidant activity of *A. genipiflora* is relatively low.

Report by Awang-Jamil et al. (2019) revealed that, *Aidia. borneensis* which is among species of the genus *Aidia*, has antioxidant activity due to its phenolic and flavonoid contents. The result of the DPPH radical scavenging assay for the isolated compounds from *A. genipiflora* revealed that all the compounds, oleanonic acid (**AG1**), 4-hydroxy cinnamic acid docosyl ester (**AG2**), β-stigmasterol and β-sitosterol (**AG3a/3b**; 2:1) and D-mannitol (**AG4**) had low radical scavenging activity with an IC_50_ range of 64–86 μg/mL compared to ascorbic acid (IC_50_ of 3.1 μg/mL). Free radicals may prolong inflammatory response of the body and cause further tissue damage (Khanna et al., 2014). Hence the antioxidant activities of the extracts and compounds of *A. genipiflora* would be beneficial in the anti-inflammatory activity of the plant.

## 5 Conclusion

The result of this research is the first report on the *in vivo* acute anti-inflammatory activity of *Aidia genipiflora* stem bark extracts in carrageenan-induced chick model and the antioxidant activity of *Aidia genipiflora* stem bark extract using DPPH radical scavenging assay. This is a scientific validation of its traditional uses in the management of inflammatory conditions. The compounds isolated from Aidia genipiflora, oleanonic acid (**AG1**), 4-hydroxy cinnamic acid docosyl ester (**AG2**), β-stigmasterol and β-sitosterol (**AG3a/3b**; 2:1) and D-mannitol (**AG4**) demonstrated potent anti-inflammatory and antioxidant activities thereby providing evidence for the plant as a source of bioactive compounds for the development of alternative or new anti-inflammatory therapy.

## Declarations

### Author contribution statement

Daniel Anokwah: Conceived and designed the experiments; Performed the experiments; Analyzed and interpreted the data; Contributed reagents, materials, analysis tools or data; Wrote the paper.

Evelyn Asante Kwatia; Benjamin Kingsley Harley: Analyzed and interpreted the data; Contributed reagents, materials, analysis tools or data; Wrote the paper.

Isaac Kingsley Amponsah; Ernest Obese: Performed the experiments.

Yakubu Jibira; Elvis Ofori Ameyaw; Robert Peter Biney: Performed the experiments; Wrote the paper.

Abraham Yeboah Mensah: Conceived and designed the experiments; Contributed reagents, materials, analysis tools or data; Wrote the paper.

### Funding statement

This research did not receive any specific grant from funding agencies in the public, commercial, or not-for-profit sectors.

### Data availability statement

Data will be made available on request.

### Declaration of interest’s statement

The authors declare no conflict of interest.

### Additional information

No additional information is available for this paper.
